# Number of Offspring and Cardiovascular Disease Risk in Men and Women: The Role of Shared Lifestyle Characteristics

**DOI:** 10.1097/EDE.0000000000000712

**Published:** 2017-09-28

**Authors:** Maria C. Magnus, Stamatina Iliodromiti, Debbie A. Lawlor, Janet M. Catov, Scott M. Nelson, Abigail Fraser

**Affiliations:** From the a MRC Integrative Epidemiology Unit at the University of Bristol, Bristol, United Kingdom; b School of Social and Community Medicine, University of Bristol, Bristol, United Kingdom; c Domain for Mental and Physical Health, Norwegian Institute of Public Health, Oslo, Norway; d School of Medicine, University of Glasgow, Glasgow, Scotland; and e Department of Obstetrics and Gynecology, University of Pittsburgh, Pittsburgh, PA.

## Abstract

**Background:**

Previous studies of the number of offspring and cardiovascular disease (CVD) report conflicting findings. We re-examined this association in both sexes to clarify the role of the cardiometabolic changes that women experience during pregnancy versus shared lifestyle characteristics.

**Methods:**

We studied 180,626 women and 133,259 men participating in the UK Biobank cohort who were free of CVD at baseline. CVD events were obtained from hospital and death registers. Analyses were conducted using Cox proportional hazards regression.

**Results:**

The incidence rates of overall CVD were six per 1000 person-years for women and nine per 1000 person-years for men. Number of children showed an association with risk of CVD among women; the adjusted HR (95% CI) was 1.2 (1.1, 1.3) for one, 1.1 (1.0, 1.2) for two, 1.2 (1.1, 1.3) for three, and 1.2 (1.1, 1.4) for four or more as compared to none. Number of children was also associated with CVD among men; the adjusted HR (95% CI) was 1.1 (1.0, 1.2) for one, 1.0 (0.96, 1.1) for two, 1.1 (1.0, 1.2) for three, and 1.1 (1.0, 1.3) for four or more as compared to none. There was no evidence of heterogeneity in the associations between sexes (*P*_interaction_ = 0.80). Number of offspring also showed similar associations with ischemic heart disease and hypertensive disorders in both sexes.

**Conclusions:**

We observed similar associations between number of offspring and CVD in both sexes. The association among women might therefore be largely explained by unobserved behavioral and lifestyle characteristics.

## Abstract 2

Supplemental Digital Content is available in the text.

The number of pregnancies a woman experiences might increase her risk of cardiovascular disease (CVD) because of the cardiometabolic stress of pregnancy.^1–3^ However, the current evidence remains inconclusive. Previous studies have reported positive,^4–6^ null,^7–11^ inverse,^12^ and “J-shaped”/nonlinear^13–17^ associations between number of offspring and CVD among women. These studies have varied in size, outcome classification, and adjustment for relevant background characteristics.

Potential nonexclusive explanations for an increased CVD risk with increasing number of offspring in women include the following: (1) adverse cardiometabolic changes that occur in pregnancy accumulate across pregnancies, resulting in later CVD^1–3^; (2) unhealthy lifestyle and/or behavior as a consequence of raising more children, which subsequently increases the risk of CVD^18^; and/or (3) individuals from lower socioeconomic backgrounds tend to have more children, partly due to delayed childbearing among those of higher socioeconomic status, and are at higher CVD risk^19,20^ (Figure 1).

**FIGURE 1.**
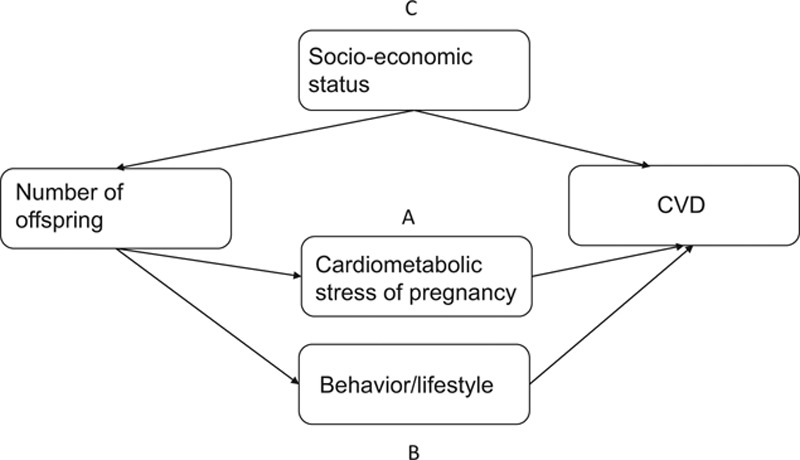
Potential mechanisms for an association between number of offspring and cardiovascular disease.

One way to evaluate whether the observed association between number of offspring and CVD observed among women is due to pregnancy per se is to compare the association between sexes. Any association observed among men would have to be explained by other factors, and men therefore serve as negative controls.^21^ Among studies that evaluated number of children/offspring and CVD risk in both sexes, some only evaluated CVD mortality,^6,15^ could not adjust for lifestyle factors^15^ and/or had limited opportunity to more closely evaluate subgroups of CVD.^5,14,17,22^

The aim of the current study was therefore to examine the association between number of offspring and CVD risk in a large population of both women and men to clarify the role of cardiometabolic changes related to pregnancy versus shared lifestyle characteristics.

## METHODS

### Study Population

**FIGURE 2.**
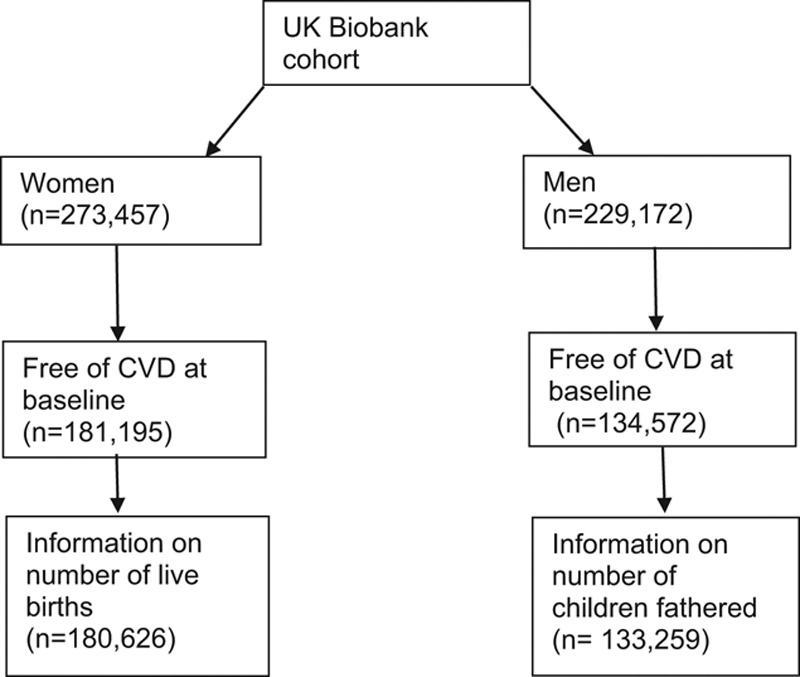
Illustration of study sample.

This study included participants in UK Biobank, including 503,325 individuals who were 40–69 years of age, recruited between 2006 and 2010 from across England, Scotland, and Wales, registered with the UK National Health Service (NHS), and living within 25 miles of one of the 22 assessment centers.^23,24^ More than 10 million individuals were invited to participate, and the participation rate was 5%. After giving consent, participants completed a questionnaire, an interview, and a physical examination. This information was linked to national hospital and death registers. The end of follow-up was 28 February 2015 for England, 16 March 2015 for Wales, and 28 October 2014 for Scotland. UK Biobank is approved by the NHS National Research Ethics Service (Ref 11/NW/0382). Information on 273,457 women and 229,172 men was available. After excluding prevalent cases and those without exposure information, 180,626 women and 133,259 men contributed to analyses (Figure 2).

### Number of Offspring

The exposure was self-reported number of live births for women and the number of children fathered for men, categorized as none, 1, 2, 3, and 4+ children. For men, there was a separate category indicating do not know/prefer not to say. We evaluated the number of live births for women, since cardiometabolic changes due to pregnancy are likely to be more pronounced if the pregnancy is longer.^3^

### Cardiovascular Disease Events

CVD events were available from hospital and death registers, coded according to the International Classification of Diseases (ICD). The primary outcome was any CVD (morbidity and mortality) (ICD-10 codes: I00–I99). Secondary outcomes included ischemic heart disease (IHD; I20–I25), cerebrovascular disease (I60–I69), hypertensive disorders (I10–I15), heart failure (I50), pulmonary heart disease and diseases of pulmonary circulation (I26–I28), and diseases of arteries, arterioles, and capillaries (I70–I79). More details are available in eTable 1 (http://links.lww.com/EDE/B230).

### Other Variables

Information on age, ethnicity (white, Asian, black, mixed/other, unknown/prefer not to answer), qualifications (college, university or other professional degree, Advance levels/Advance Subsidiary levels or equivalent, Ordinary levels/General Certificate of Secondary Educations or equivalent, Certificate of Secondary Educations or equivalent, National Vocational Qualifications, Higher National Diploma, Higher National Certificate or equivalent, other, prefer not to answer), Townsend deprivation index, and household income in pounds (<18,000, 18,000–30,999, 31,000–51,999, 52,000–100,000, >100,000, do not know, prefer not to answer) was obtained by self-report. As were smoking (never, former, current, prefer not to answer), alcohol intake (daily or almost daily, three or four times a week, once or twice a week, one to three times a month, special occasions only, never, prefer not to answer), physical activity calculated into estimated metabolic equivalents/min/wk (METS), family history of CVD (yes/no), and diabetes (no, yes, do not know/prefer not to answer). Questions on physical activity included both type and duration of physical activity (including walking, “do-it-yourself” home activities, moderate and vigorous physical activity, strenuous sports, etc.). METS were calculated based on answers to questions on frequency and duration of different moderate to vigorous physical activities scored as defined in the International Physical Activity Questionnaire.^25^ Weight and height, used to calculate body mass index (BMI; kg/m2), and systolic and diastolic blood pressure (average of two measurements), were measured by nurses.

### Statistical Analysis

We plotted incidence rates of overall CVD against number of children to identify the category which had the lowest risk and used this as the reference. We then evaluated the associations using Cox proportional hazard regression, reporting hazard ratios (HRs), and 95% confidence intervals (CIs). Contributions to risk were censored at the first event, death from any other cause, or end of the follow-up. The underlying time scale for the Cox model was calendar time. We also conducted a sensitivity analysis using age as the time axis. The proportional hazards assumption was evaluated by the Schoenfeld residuals. Model 1 was adjusted for age and model 2 was adjusted for age, ethnicity, qualifications, income, Townsend deprivation index, family history of CVD, smoking, alcohol intake, physical activity, BMI, diabetes, and blood pressure. To test for a nonlinear association, we used a likelihood ratio test comparing the models including number of offspring categorically versus continuously. Differences between sexes were tested by comparing the models with and without the four interaction terms using a likelihood ratio test.

To compare similarly adjusted associations for men and women, we limited the adjustment to characteristics shared between sexes. However, we conducted additional analyses of the associaiton between number of offspring and CVD among women further adjusting for age at menarche (continuous), menopausal status (no, yes, do not know due to hysterectomy, and do not know due to other reasons), hormone replacement therapy (yes vs. no), and oral contraceptive use (yes vs. no). Secondary analyses evaluated the associations of number of offspring with subcategories of CVD. Results from the likelihood ratio tests presented for interactions and linearity were from the fully adjusted model.

We conducted a number of sensitivity analyses. We explored the association between parity (including live births and stillbirths) and CVD risk among women. We evaluated multiplicative interactions by age and country on the association between number of offspring and CVD in both sexes, since individuals in the youngest age categories might have had additional children after baseline and registration practices in national registries might vary between countries, by comparing a model with the interactions to a model with no interaction terms using a likelihood ration test. Age was categorized according to four approximately equal age groups (<49, 49–55, 56–62, and 63 or over), which were also used in the interaction test. We also explored a stratified analysis by year of birth (<1947, 1947–1952, 1953–1959, and 1960 and later), to evaluate a possible cohort effect. Because of the low participation rate, we examined the associations of established CVD risk factors (age, alcohol, smoking, BMI, blood pressure, and diabetes) in UK Biobank, to determine the likelihood of selection bias. As an additional evaluation of a potential selection bias, we examined the association between number of offspring and all-cause mortality.

All results presented are from complete case analyses. All analyses were conducted using Stata version 14 (Statacorp, College Station, TX).

## RESULTS

The distribution of characteristics by sex is given in Table 1, while the distribution of characteristics by number of offspring for each sex is given in eTables 2 and 3 (http://links.lww.com/EDE/B230). Individuals who reported having no children were younger, more likely to have higher education, had lower BMI, had lower blood pressure, were more likely to have diabetes, and were less likely to smoke (eTables 2 and 3; http://links.lww.com/EDE/B230). Median follow-up time was 6.0 years (interquartile range: 5.3–6.6). The incidence rates of overall CVD were six per 1000 person-years for women and nine per 1000 person-years for men (eTable 4; http://links.lww.com/EDE/B230).

**TABLE 1.**
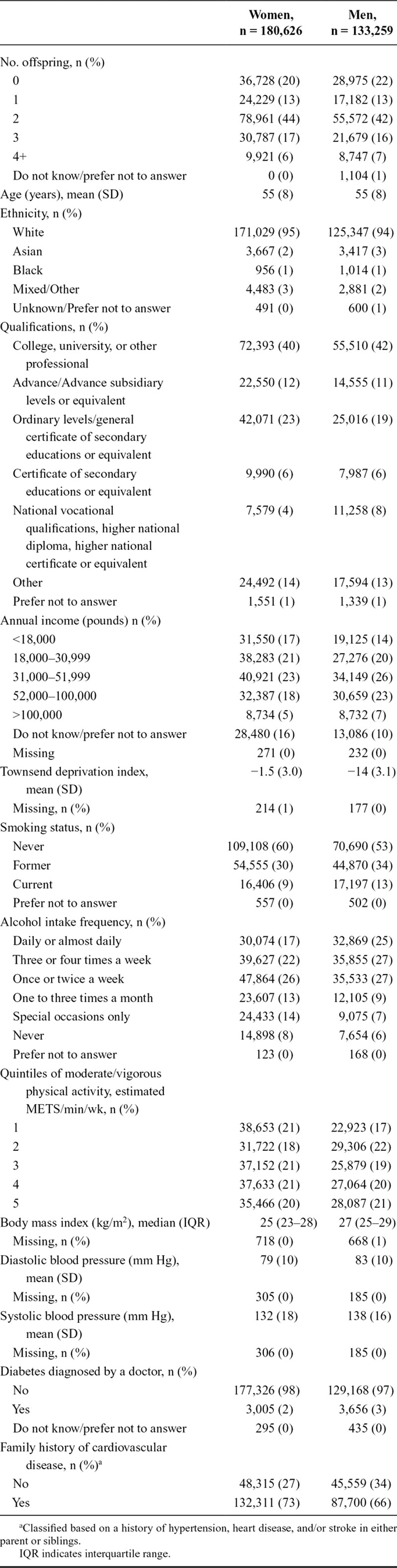
Distribution of Characteristics

### The Association Between Number of Offspring and Risk of CVD Among Women

**FIGURE 3.**
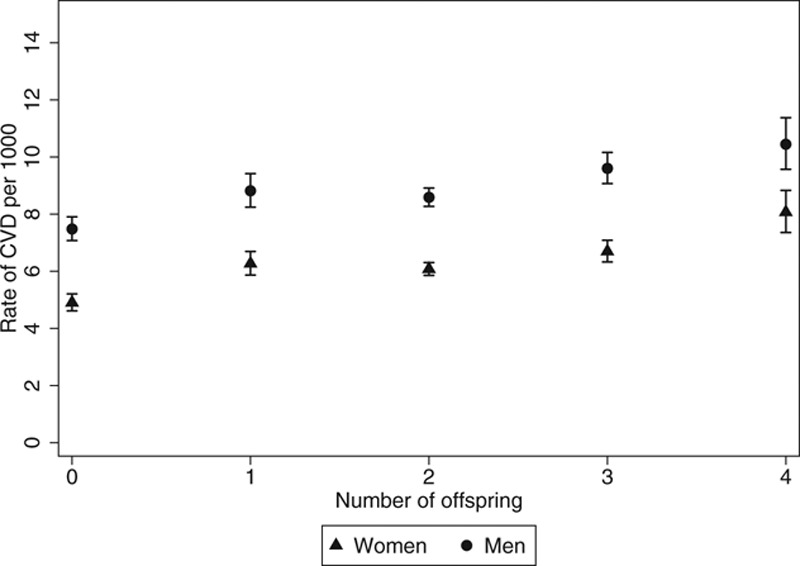
Incidence rates of cardiovascular disease by number of offspring.

The rate of CVD was lowest among women with no live births (Figure 3), and we used this group as the reference. Number of live births showed a nonlinear association with CVD risk (*P*_nonlinearity_ = 0.05), as the risk among women with at least one live birth was lowest among those with two (Table 2). Multivariable adjustment caused only minor changes to the observed associations (Table 2). Number of live births was also associated with IHD and hypertensive disorders, with statistical evidence of a nonlinear association for hypertensive disorders (*P*_nonlinearity_ < 0.001) (Table 2). There was no association of number of live births with cerebrovascular disease, heart failure, or pulmonary heart disease and diseases of pulmonary circulation. Women with 2–3 live births, but not women with four or more live births, had a reduced risk of disease of the arteries, arterioles, and capillaries (*P*_nonlinearity_ = 0.05).

**TABLE 2.**
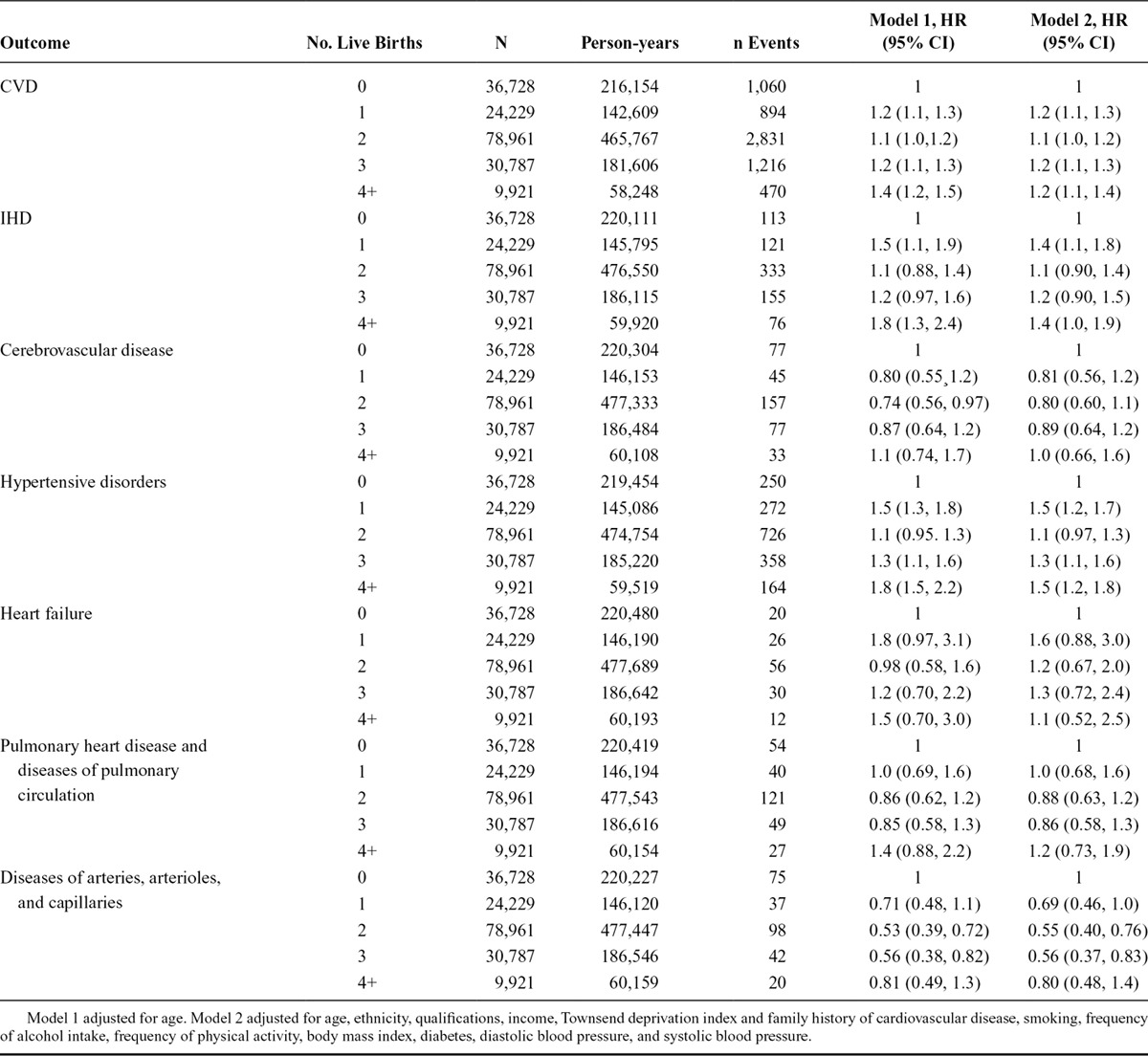
The Association Between Number of Live Births and Cardiovascular Disease Among Women

The proportional hazards assumption was violated for a few of the covariates in the models of overall CVD and IHD, but including time-varying covariates did not change the association of interest (results available upon request). Adjustment for reproductive health indicators did not change the associations (eTable 5; http://links.lww.com/EDE/B230), nor did using parity as the exposure (eTable 6; http://links.lww.com/EDE/B230).

### The Association Between Number of Children Fathered and Risk of CVD Among Men

The risk of CVD was lowest among men who had not fathered children (Figure 3). The risk of CVD among those with any children was lowest among those with two children (Table 3). Similar patterns of associations were observed for IHD and hypertensive disorders (Table 3). There were no associations with cerebrovascular disease, heart failure, pulmonary heart disease and disease of pulmonary circulation, or diseases of arteries, arterioles, and capillaries.

**TABLE 3.**
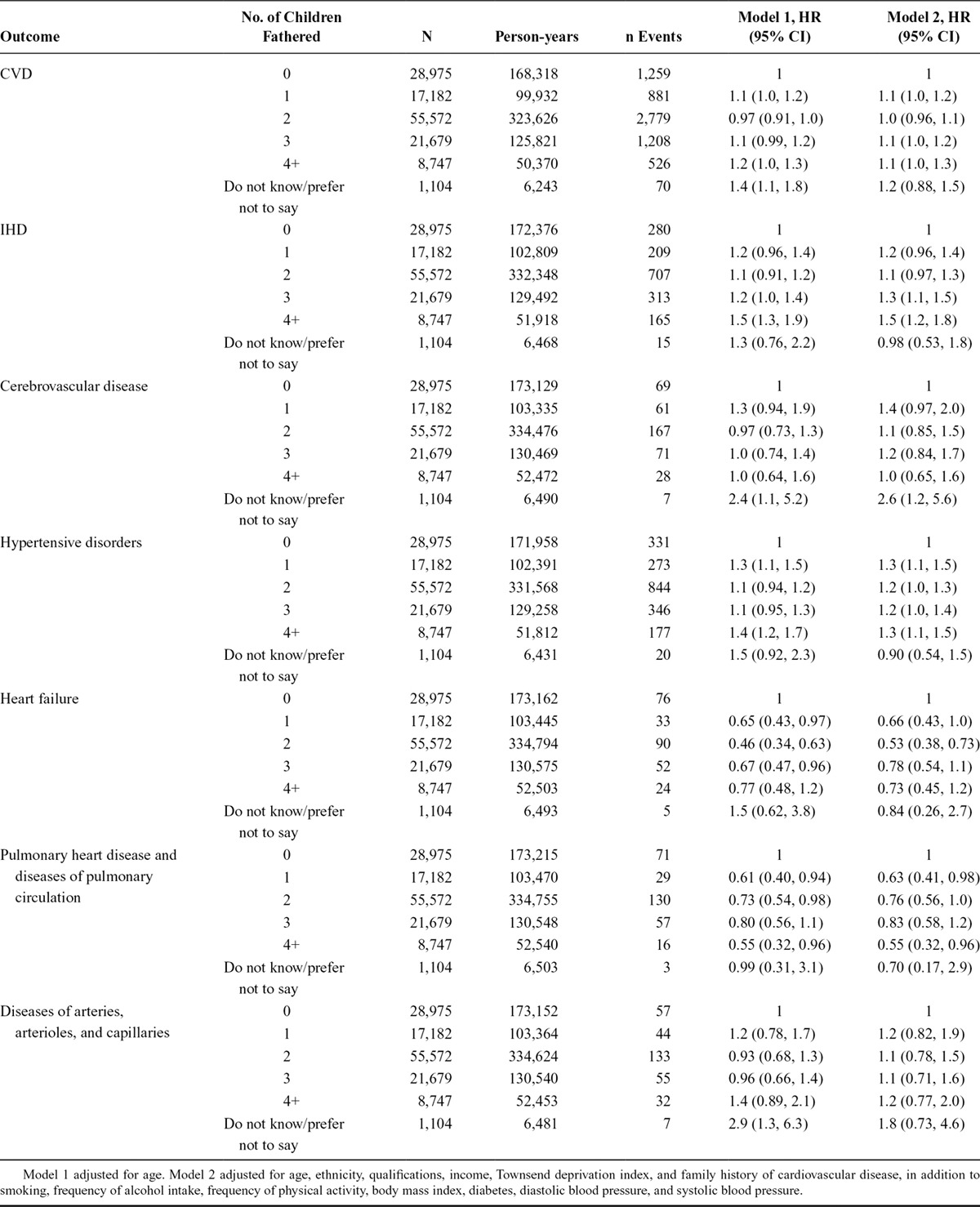
The Association Between the Number of Children Fathered and Cardiovascular Disease Among Men

There was no evidence of nonlinear associations with any of the CVD outcomes (*P*_nonlinearity_ > 0.11), except for heart failure (*P*_nonlinearity_ = 0.01). There was no evidence of any violation of the proportional hazards assumption.

### Comparison of the Association Between Number of Children and Overall CVD Between Sexes

The associations between number of offspring and CVD risk were similar in both sexes, with the nadir of risk among those with no children, and the risk among those with at least one child was lowest among those with two (Figure 2). The magnitude of the association tended to be greater among women, but with no strong evidence of a dose–response relationship (Tables 2 and 3). Finally, there was no statistical evidence of heterogeneity in the associations between the sexes (overall *P*_interaction_ = 0.80). The point estimates of the four interaction terms ranged from 0.96 to 1.0 with relatively narrow confidence intervals (all *P* > 0.53).

### Additional Analyses

Additional adjustment for whether the participants were currently living with a partner/spouse did not change the associations (results available upon request). There was no strong evidence of interaction for the association between number of offspring and CVD by country (eTables 7 and 8; http://links.lww.com/EDE/B230). There were some tendencies toward associations of a greater magnitude among the two youngest age categories (eTables 9 and 10; http://links.lww.com/EDE/B230), but no strong evidence of heterogeneity based on the test for interaction. The stratified analyses by year of birth (eTables 11 and 12; http://links.lww.com/EDE/B230) largely indicated similar associations as the stratified analysis by age at recruitment. Due to the relatively short recruitment period, the correlation coefficient between age at recruitment and year of birth was 0.99, and both could therefore not be included in the multivariable analysis as continuous covariates. The sensitivity analysis using age as the time axis yielded associations of a slightly greater magnitude, but the results were overall similar (eTable 13; http://links.lww.com/EDE/B230). Associations of established risk factors with CVD were also as expected (eTable 14; http://links.lww.com/EDE/B230). There was a modest increased risk of all-cause mortality among those who had never had offspring compared to those who had at least one (eTable 15; http://links.lww.com/EDE/B230).

## DISCUSSION

In this large-scale, prospective cohort study, the association between number of offspring and risk of overall CVD, IHD, and hypertensive disorders was similar in both sexes, with the nadir of risk among those with no children. In those with children, the risk was lowest among participants with two children. Furthermore, there was some evidence that the number of offspring was associated with reduced risk of diseases of arteries, arterioles, and capillaries among women and with reduced risk of heart failure among men.

Strengths of our study include the comparison of associations in both sexes, its size, its prospective design, linkage to hospital registers, and the ability to more closely evaluate subgroups of CVD. The study also has limitations. Individuals who were older at the time of recruitment might be underlying healthier than those who were younger since participation was contingent on surviving until recruitment. Since the number of offspring increased with age at baseline, this would likely attenuate our associations toward the null. Despite the large sample size, due to the short follow-up time, estimates for women in CVD subgroups were imprecise compared to those obtained for men due to the lower disease incidence in women. The low participation rate might also influence the generalizability of our findings, but not necessarily the internal validity.^26–28^ Notably, the average number of offspring in UK Biobank is the same as the national average,^29^ the associations of known risk factors with CVD were in the expected direction and magnitude,^30^ and the association between number of offspring and all-cause mortality was also as expected.^31^ We acknowledge the potential limitations of using registries to obtain outcome information, as they rely on the accurate registration practices of clinicians.^32^ Measurements of covariates available in UK Biobank were arguably crude and largely based on self-report. This is an expected limitation of such a large-scale cohort. Since the adjustment for available measures of potential confounders caused a substantial attenuation of the observed associations, we cannot exclude the possibility that the remaining associations might be explained by residual confounding. Information on marital status was unavailable, as was whether the offspring were attained by multiple partners and underlying reasons for childbearing or childlessness. Furthermore, participants might have had additional offspring after baseline, and our exposure could therefore be underestimated. The fertility rate among women in the United Kingdom 40 years old and older was approximately 15 per 1,000 in 2015.^33^ A small group of UK Biobank participants (10,392 women) were invited for a follow-up visit in 2012–2013. Thirty-five women out of those (<0.5%) had a further pregnancy since the time of recruitment. The misclassification is therefore likely to be minimal. Finally, we did not have information on pregnancy complications, and the associations observed among women are therefore to be interpreted as total associations including the potential influence of pregnancy complications.

Several studies report increased risk of CVD with increasing number of offspring among women,^4–6^ while others reported no association,^7–11^ an inverse association,^12^ while yet other studies suggest a J-shaped/nonlinear association.^13–16^ A potential explanation for previous studies reporting no association might be due to moderate power (n = 867–19,688),^8,10,11^ the evaluation of a largely high socioeconomic population^7^ or ethnic differences in the association.^9^ The magnitude of the associations reported in previous studies varies, as they use different reference categories and had a different distribution in number of offspring.

Some previous studies evaluated the association between number of offspring and CVD in both sexes.^5,6,14,15,22^ Analyses of the Framingham and the first National Health and Nutrition Examination Survey National Epidemiologic Follow-up Study (NHEFS) studies (associations for men and women published separately)^5,22^ in addition to the Dubbo Australian cohort^6^ found linear positive associations in women and no association among men. In contrast, the British Regional Heart and Women’s Heart and Health Studies^14^ and the Israel Longitudinal Mortality Study II^15^ indicated nonlinear associations between number of children and IHD morbidity in both sexes with a nadir of risk among those who had two children. Finally, the China Kadoorie Biobank recently reported a similar association between the sexes with the nadir of risk among those with one child.^17^

Most previous studies only evaluated CVD mortality.^6,8,9,11,12,15,16^ Our study contributes new information because we included both CVD morbidity and mortality and more closely evaluated CVD subgroups. In some CVD subcategories—heart failure and pulmonary heart disease and diseases of pulmonary circulation in men and disease of arteries, arterioles, and capillaries in women—we found associations in the opposite direction to what was expected, assuming that all of these outcomes have a shared underlying etiology. However, the confidence intervals were wide as these outcomes were rare. Such nuances between CVD subgroups had not been available in previous studies that only evaluated overall CVD, IHD, and/or cerebrovascular disease.

We hypothesized that the association between number of offspring and CVD among women could reflect pregnancy-related cardiometabolic changes (Figure 1, path A). Since the associations were so similar in both sexes, it is unlikely that this explains much of the observed association among women.

Another explanation is adverse lifestyle related to having more children, which may have consequence for later CVD risk (Figure 1, path B).^18^ Lower socioeconomic status is associated with both larger family size and adverse lifestyle factors that have a potential influence on the risk of CVD (Figure 1, path C).^19^ We adjusted for multiple measures of socioeconomic position and lifestyle factors, but we cannot rule out residual confounding. The similar associations in both sexes suggests that the association observed among women largely reflects such shared characteristics. This is under the assumption that any such unobserved confounding would largely be similarly distributed between sexes. The reduction in conventional CVD risk factors therefore seems to be the best approach to reduce the differential burden of CVD by number of offspring.

Several studies indicate that the lowest risk of CVD is observed among those with two births,^13–15^ which has been attributed to an influence of subfertility.^34,35^ We did not see a J-shaped association between number of offspring and CVD risk; the lowest CVD risk was seen among women with no children. In contrast to some of these previous studies, our findings therefore seem to suggest that infertility may not have a notable association with future CVD risk in this cohort and/or is diluted by low risk in women who are voluntarily childless. This is despite the fact that previous studies observed similar characteristics of highly educated and health conscious individuals among those with no children as was observed in UK Biobank.^5,14^

The total fertility rate peaked in the United Kingdom in the early 1960s, with a fertility rate of 2.9 per woman, and gradually decreased to around 1.7 in the late 1970s.^29^ Since the 1980s, the fertility rate has been relatively stable between 1.6 and 1.9.^29^ The participants in UK Biobank were born between 1934 and 1971. Estimates of the associations between number of offspring and CVD were of greater magnitude among individuals born after the 1960s, but there was no strong evidence of heterogeneity by age at recruitment or year of birth.

In conclusion, we observed similar associations between number of offspring and CVD in both sexes. The association between number of offspring and CVD among women might therefore be largely explained by unobserved behavioral and lifestyle characteristics.

## ACKNOWLEDGMENTS

This research has been conducted using the UK Biobank Resource. We are grateful to all the participants in UK Biobank.

## Supplementary Material
