# Supplementary material for: Number of Offspring and Cardiovascular Disease Risk in Men and Women: The Role of Shared Lifestyle Characteristics
Source: Epidemiology. 2017 Sep 28;28(6):880–8. doi: 10.1097/EDE.0000000000000712 (PMC5625954; doi:10.1097/EDE.0000000000000712)
Supplement: Supplementary file 1 [file ede-28-880-s001.pdf]

Online Supplementary Material

eTable 1 Description of ICD 10 codes included in the different outcome definitions

Cardiovascular disease outcome	ICD 10 codes	Description
Overall CVD	I00-I99	I00-I02 Acute rheumatic fever<br>I05-I09 Chronic rheumatic heart diseases<br>I10-I15 Hypertensive diseases<br>I20-I25 Ischemic heart diseases<br>I26-I28 Pulmonary heart disease and diseases of pulmonary circulation<br>I30-I52 Other forms of heart disease<br>I60-I69 Cerebrovascular diseases<br>I70-I79 Diseases of arteries, arterioles and capillaries<br>I80-I89 Diseases of veins, lymphatic vessels and lymph nodes, not elsewhere classified<br>I95-I99 Other and unspecified disorders of the circulatory system
IHD	I20-I25	I20 Angina pectoris<br>I21 Acute myocardial infarction<br>I22 Subsequent myocardial infarction<br>I23 Certain current complications following acute myocardial infarction<br>I24 Other acute ischemic heart diseases<br>I25 Chronic ischemic heart disease
Cerebrovascular disease	I60-I69	I60 Subarachnoid haemorrhage<br>I61 Intracerebral haemorrhage<br>I62 Other nontraumatic intracranial haemorrhage<br>I63 Cerebral infarction<br>I64 Stroke, not specified as haemorrhage or infarction<br>I65 Occlusion and stenosis of precerebral arteries, not resulting in cerebral infarction<br>I66 Occlusion and stenosis of cerebral arteries, not resulting in cerebral infarction<br>I67 Other cerebrovascular diseases<br>I68 Cerebrovascular disorders in diseases classified elsewhere<br>I69 Sequelae of cerebrovascular disease
Hypertensive disorders	I10-I15	I10 Essential (primary) hypertension<br>I11 Hypertensive heart disease<br>I12 Hypertensive renal disease<br>I13 Hypertensive heart and renal disease<br>I15 Secondary hypertension
Heart failure	I50	
Pulmonary heart disease and diseases of pulmonary circulation	I26-I28	I26 Pulmonary embolism<br>I27 Other pulmonary heart diseases<br>I28 Other diseases of pulmonary vessels
Diseases of arteries, arterioles and capillaries	I70-I79	I70 Atherosclerosis<br>I71 Aortic aneurysm and dissection<br>I72 Other aneurysm and dissection<br>I73 Other peripheral vascular diseases<br>I74 Arterial embolism and thrombosis<br>I77 Other disorders of arteries and arterioles<br>I78 Diseases of capillaries<br>I79 Disorders of arteries, arterioles and capillaries in diseases classified elsewhere

eTable 2 The Distribution of Characteristics by the Number of Live Births Among Women

	Live births				
Characteristics	0<br>n= 36,728	1<br>n= 24,229	2<br>n= 78,961	3<br>n= 30,787	4 or more<br>n= 9,921
Age, years [mean(SD)]	53 (8)	54 (8)	56 (8)	56 (8)	57 (8)
Ethnicity [n(%)]					
White	34,787 (95)	22,612 (93)	75,744 (96)	29,076 (94)	8,810 (89)
Asian	626 (2)	545 (2)	1,378 (2)	733 (2)	385 (4)
Black	115 (0)	144 (1)	255 (0)	205 (1)	237 (2)
Mixed/Other	1,080 (3)	841 (4)	1,415 (2)	692 (2)	455 (5)
Unknown/Prefer not to answer	120 (0)	87 (0)	169 (0)	81 (0)	34 (0)
Qualifications [n(%)]					
College, university or other professional	18,687 (51)	9,172 (38)	29,704 (38)	11,570 (38)	3,260 (33)
A levels/AS levels or equivalent	4,997 (14)	2,984 (12)	9,814 (12)	3,658 (12)	1,097 (11)
O levels/GCSEs or equivalent	7,532 (21)	5,977 (25)	19,500 (25)	7,082 (23)	1,980 (20)
CSEs or equivalent	1,616 (4)	1,596 (7)	4,567 (6)	1,704 (6)	507 (5)
NVQ, HND, HNC or equivalent	1,069 (3)	1,062 (4)	3,357 (4)	1,479 (5)	612 (6)
Other	2,602 (7)	3,209 (13)	11,361 (14)	5,018 (16)	2,302 (23)
Prefer not to answer	225 (1)	229 (1)	658 (1)	276 (1)	163 (2)
Income, pounds [n(%)]					
Less than 18,000	6,111 (17)	4,586 (19)	12,709 (16)	5,605 (18)	2,539 (26)
18,000-31,000	8,377 (23)	5,256 (22)	16,517 (21)	6,182 (20)	1,951 (20)
31,000-52,000	8,959 (24)	5,757 (24)	18,004 (23)	6,488 (21)	1,713 (17)
52,000-100,000	6,731 (18)	4,137 (17)	14,807 (19)	5,428 (18)	1,284 (13)
Greater than 100,000	1,692 (5)	921 (4)	3,881 (5)	1,786 (6)	454 (5)
Do not know/prefer not to answer	4,803 (13)	3,530 (15)	12,955 (16)	5,252 (17)	1,940 (20)
Missing	55 (0)	42 (0)	88 (0)	46 (0)	40 (0)
Townsend Deprivation Index [mean(SD)]	-0.8 (3.1)	-1.2 (3.1)	-1.9 (2.7)	-1.6 (2.9)	-0.6 (3.4)
Missing[n(%)]	42 (0)	40 (0)	99 (0)	24 (0)	9 (0)
Smoking status [n(%)]					
Never	22,736 (62)	13,491 (56)	48,673 (62)	18,471 (60)	5,737 (58)
Former	10,302 (28)	7,834 (32)	24,000 (30)	9,426 (31)	2,993 (30)
Current	3,605 (10)	2,827 (12)	6,054 (8)	2,776 (9)	1,144 (12)
Prefer not to answer	85 (0)	77 (0)	234 (0)	114 (0)	47 (1)
Alcohol intake frequency [n(%)]					
Daily or almost daily	6,977 (19)	3,943 (16)	12,988 (17)	4,880 (16)	1,286 (13)
Three or four times a week	8,097 (22)	4,972 (21)	18,285 (23)	6,537 (21)	1,736 (18)
Once or twice a week	9,255 (25)	6,391 (26)	21,814 (28)	8,162 (27)	2,242 (23)
One to three times a month	4,810 (13)	3,424 (14)	10,171 (13)	3,954 (13)	1,248 (13)
Special occasions only	4,619 (13)	3,483 (14)	10,064 (13)	4,412 (14)	1,855 (19)
Never	2,941 (8)	1,995 (8)	5,595 (7)	2,829 (9)	1,538 (16)
Prefer not to answer	29 (0)	21 (0)	44 (0)	13 (0)	16 (0)
Quintiles of moderate/vigorous physical activity METS/min/week [n(%)]					
1	7,447 (20)	5,401 (22)	16,720 (21)	6,608 (22)	2,477 (25)
2	7,046 (19)	4,435 (18)	13,535 (17)	5,110 (17)	1,596 (16)
3	7,649 (21)	4,799 (20)	16,546 (21)	6,238 (20)	1,920 (19)
4	7,771 (21)	4,967 (21)	16,648 (21)	6,404 (21)	1,843 (19)
5	6,815 (19)	4,627 (19)	15,512 (20)	6,427 (21)	2,085 (21)
Body-mass index (kg/m^2) [median(IQR)][a]	25 (23, 28)	25 (23, 29)	25 (23, 28)	26 (23, 29)	27 (24, 30)
Missing	175 (1)	108 (0)	265 (0)	105 (0)	65 (1)
Diastolic blood pressure (mmHG) [mean(SD)]	79 (10)	79 (10)	79 (9)	79 (10)	80 (10)
Missing	77 (0)	53 (0)	116 (0)	37 (0)	22 (0)
Systolic blood pressure (mmHG) [mean(SD)]	130 (17)	131 (18)	132 (18)	133 (18)	133 (18)
Missing	77 (0)	53 (0)	116 (0)	37 (0)	23 (0)

Diabetes diagnosed by a doctor [n(%)]					
No	36,175 (99)	23,774 (98)	77,662 (98)	30,123 (98)	9,592 (97)
Yes	513 (1)	421 (2)	1,179 (2)	607 (2)	285 (3)
Do not know/prefer not to answer	40 (0.1)	34 (0.1)	120 (0.2)	57 (0.2)	44 (0.4)
Family history of cardiovascular disease [n(%)][b]					
No	9,641 (26)	6,630 (27)	20,810 (26)	8,422 (27)	2,812 (28)
Yes	27,087 (74)	17,599 (73)	58,151 (74)	22,365 (73)	7,109 (72)

IQR: interquartile range; METS: metabolic equivalents; SD: standard deviation

P-values for trends <0.001 for all comparisons

[a] Median and interquartile range

[b] Classified based on a history of hypertension, heart disease and/or stroke in either parent or siblings.

eTable 3 Distribution of Characteristics by Number of Children Fathered among Men

	Number of children fathered					
Characteristics	0<br><br>n= 28,975	1<br><br>n= 17,182	2<br><br>n= 55,572	3<br><br>n= 21,679	4 or more<br><br>n= 8,747	Do not know/prefer not to answer<br>n= 1,104
Age, years [mean(SD)]	53 (8)	54 (8)	56 (8)	56 (8)	56 (8)	54 (8)
Ethnicity [n(%)]						
White	27,718 (96)	16,231 (95)	53,051 (96)	20,176 (93)	7,307 (84)	864 (78)
Asian	423 (2)	369 (2)	1,279 (2)	698 (3)	565 (7)	83 (8)
Black	102 (0)	107 (1)	218 (0)	248 (1)	318 (4)	21 (2)
Mixed/Other	588 (2)	398 (2)	855 (2)	479 (2)	496 (6)	65 (6)
Unknown/Prefer not to answer	144 (1)	77 (1)	169 (0)	78 (0)	61 (1)	71 (6)
Qualifications [n(%)]						
College, university or other professional	12,959 (45)	6,573 (38)	22,974 (41)	9,337 (43)	3,357 (38)	310 (28)
A levels/AS levels or equivalent	3,623 (13)	1,848 (11)	6,030 (11)	2,177 (10)	794 (9)	83 (8)
O levels/GCSEs or equivalent	5,233 (18)	3,552 (21)	10,794 (19)	3,848 (18)	1,429 (16)	160 (15)
CSEs or equivalent	1,751 (6)	1,229 (7)	3,214 (6)	1,185 (6)	534 (6)	74 (7)
NVQ, HND, HNC or equivalent	2,063 (7)	1,490 (9)	5,049 (9)	1,837 (9)	721 (8)	98 (9)
Other	3,101 (11)	2,312 (14)	7,038 (13)	3,093 (14)	1,785 (20)	265 (24)
Prefer not to answer	245 (1)	178 (1)	473 (1)	202 (1)	127 (2)	114 (10)
Income, pounds [n(%)]						
Less than 18,000	5,759 (20)	2,390 (14)	6,242 (11)	2,715 (13)	1,724 (20)	295 (27)
18,000-31,000	6,321 (22)	3,595 (21)	11,185 (20)	4,255 (20)	1,732 (20)	188 (17)
31,000-52,000	6,866 (24)	4,738 (28)	14,942 (27)	5,594 (26)	1,880 (22)	129 (12)
52,000-100,000	5,515 (19)	3,882 (23)	14,136 (25)	5,346 (25)	1,687 (19)	93 (8)
Greater than 100,000	1,403 (5)	938 (6)	3,884 (7)	1,816 (8)	673 (8)	18 (2)
Do not know/prefer not to answer	3,030 (11)	1,613 (9)	5,126 (9)	1,926 (9)	1,020 (12)	371 (34)
Missing	81 (0)	26 (0)	57 (0)	27 (0)	31 (0)	10 (1)
Townsend Deprivation Index [mean(SD)]	-0.5 (3.3)	-1.3 (3.1)	-2.0 (2.7)	-1.6 (2.9)	-0.5 (3.5)	0.8 (3.6)
Missing [n(%)]	40 (0)	28 (0)	73 (0)	23 (0)	13 (0)	0 (0)
Smoking status [n(%)]						
Never	16,530 (57)	8,717 (51)	29,733 (54)	11,230 (52)	4,051 (46)	429 (39)
Former	8,257 (29)	5,849 (34)	19,653 (35)	7,682 (35)	3,104 (36)	325 (29)
Current	4,104 (14)	2,555 (15)	6,015 (11)	2,698 (13)	1,546 (18)	279 (25)
Prefer not to answer	84 (0)	61 (0)	171 (0)	69 (0)	46 (1)	71 (6)

Alcohol intake frequency [n(%)]						
Daily or almost daily	6,802 (24)	4,176 (24)	14,125 (25)	5,543 (26)	2,017 (23)	206 (19)
Three or four times a week	7,366 (25)	4,478 (26)	15,967 (29)	5,881 (27)	1,964 (23)	199 (18)
Once or twice a week	7,620 (26)	4,703 (27)	15,178 (27)	5,737 (27)	2,053 (24)	242 (22)
One to three times a month	2,871 (10)	1,715 (10)	4,754 (9)	1,847 (9)	804 (9)	114 (10)
Special occasions only	2,318 (8)	1,182 (7)	3,236 (6)	1,392 (6)	808 (9)	139 (13)
Never	1,963 (7)	914 (5)	2,287 (4)	1,267 (6)	1,086 (12)	137 (12)
Prefer not to answer	35 (0)	14 (0)	25 (0)	12 (0)	15 (0)	67 (6)
Quintiles of moderate/vigorous physical activity as estimated METS/min/week [n(%)]						
1	5,540 (19)	3,025 (18)	8,764 (16)	3,471 (16)	1,743 (20)	380 (34)
2	6,460 (22)	3,717 (22)	12,226 (22)	4,813 (22)	1,903 (22)	187 (17)
3	5,398 (19)	3,257 (19)	11,230 (20)	4,282 (20)	1,547 (18)	165 (15)
4	5,692 (20)	3,427 (20)	11,778 (21)	4,402 (20)	1,603 (18)	162 (15)
5	5,885 (20)	3,756 (22)	11,574 (21)	4,711 (22)	1,951 (22)	210 (19)
Body-mass index (kg/m^2) [median(IQR)] [a]	26 (24, 29)	27 (25, 29)	27 (25, 29)	27 (25, 29)	27 (25, 30)	27 (24, 30)
Missing [n(%)]	148 (1)	75 (0)	223 (0)	114 (1)	73 (1)	35 (3)
Diastolic blood pressure (mmHG) [mean(SD)]	83 (10)	83 (10)	83 (9)	83 (9)	83 (10)	83 (10)
Missing [n(%)]	37 (0)	18 (0)	63 (0)	33 (0)	18 (0)	16 (1)
Systolic blood pressure (mmHG) [mean(SD)]	137 (16)	138 (16)	139 (16)	138 (16)	139 (17)	138 (17)
Missing [n(%)]	37 (0)	18 (0)	63 (0)	33 (0.2)	18 (0)	16 (1)
Diabetes diagnosed by a doctor [n(%)]						
No	28,087 (97)	16,680 (97)	54,148 (97)	20,996 (97)	8,313 (95)	944 (86)
Yes	796 (3)	456 (3)	1,339 (3)	633 (3)	389 (5)	43 (4)
Do not know/prefer not to answer	92 (0)	46 (0)	85 (0)	50 (0)	45 (1)	117 (11)
Family history of cardiovascular disease [n(%)] [b]						
No	9,714 (34)	5,969 (35)	18,784 (34)	7,406 (34)	3,202 (37)	484 (44)
Yes	19,261 (67)	11,213 (65)	36,788 (66)	14,273 (66)	5,545 (63)	620 (56)

IQR: interquartile range; METS: metabolic equivalents; SD: standard deviation

P-values for trends <0.001 for all comparisons

[a] Median and interquartile range

[b] Classified based on a history of hypertension, heart disease and/or stroke in either parent or siblings.

eTable 4 Incidence Rates of Cardiovascular Disease among Women and Men

Cardiovascular disease outcome	ICD 10	Women				Men			
	codes	N	Events	Person years	Incidence rate per 1000	N	Events	Person years	Incidence rate per 1000
CVD	I00-I99	180,626	6,471	1,064,386	6.1	133,259	6,723	774,310	8.8
IHD	I20-I25	180,626	798	1,088,491	0.7	133,259	1,689	795,412	2.1
Cerebrovascular disease	I60-I69	180,626	389	1,090,383	0.4	133,259	403	800,371	0.5
Hypertensive disorders	I10-I15	180,626	1,770	1,084,033	1.6	133,259	1,991	793,419	2.5
Heart failure	I50	180,626	144	1,091,194	0.1	133,259	280	800,973	0.3
Pulmonary heart disease and diseases of pulmonary circulation	I26-I28	180,626	291	1,090,927	0.3	133,259	306	801,031	0.4
Diseases of arteries, arterioles and capillaries	I70-I79	180,626	272	1,090,498	0.2	133,259	328	800,613	0.4

CVD: cardiovascular disease; IHD: ischemic heart disease.

eTable 5 The Association Between the Number of Live Births with Cardiovascular Disease Among Women

Cardiovascular disease outcome	Number of live births	N	Person years	n events	Model 1 HR (95% CI)	Model 2 HR (95% CI)	Model 3 HR (95% CI)
CVD	0	36,728	216,154	1,060	1	1	1
	1	24,229	142,609	894	1.2 (1.1, 1.3)	1.2 (1.1, 1.3)	1.2 (1.1, 1.3)
	2	78,961	465,767	2,831	1.1 (1.0,1.2)	1.1 (1.0, 1.2)	1.1 (1.0, 1.2)
	3	30,787	181,606	1,216	1.2 (1.1, 1.3)	1.2 (1.1, 1.3)	1.1 (1.0, 1.2)
	4+	9,921	58,248	470	1.4 (1.2, 1.5)	1.2 (1.1, 1.4)	1.2 (1.1, 1.4)
IHD	0	36,728	220,111	113	1	1	1
	1	24,229	145,795	121	1.5 (1.1, 1.9)	1.4 (1.1, 1.8)	1.3 (1.0, 1.7)
	2	78,961	476,550	333	1.1 (0.88, 1.4)	1.1 (0.90, 1.4)	1.1 (0.88, 1.4)
	3	30,787	186,115	155	1.2 (0.97, 1.6)	1.2 (0.90, 1.5)	1.1 (0.85, 1.4)
	4+	9,921	59,920	76	1.8 (1.3, 2.4)	1.4 (1.0, 1.9)	1.4 (1.0, 1.9)
Cerebrovascular disease	0	36,728	220,304	77	1	1	1
	1	24,229	146,153	45	0.80 (0.55¸1.2)	0.81 (0.56, 1.2)	0.81 (0.55, 1.2)
	2	78,961	477,333	157	0.74 (0.56, 0.97)	0.80 (0.60, 1.1)	0.78 (0.57, 1.1)
	3	30,787	186,484	77	0.87 (0.64, 1.2)	0.89 (0.64, 1.2)	0.92 (0.65, 1.3)
	4+	9,921	60,108	33	1.1 (0.74, 1.7)	1.0 (0.66, 1.6)	1.1 (0.68, 1.6)
Hypertensive disorders	0	36,728	219,454	250	1	1	1
	1	24,229	145,086	272	1.5 (1.3, 1.8)	1.5 (1.2, 1.7)	1.5 (1.2, 1.7)
	2	78,961	474,754	726	1.1 (0.95. 1.3)	1.1 (0.97, 1.3)	1.1 (0.96, 1.3)
	3	30,787	185,220	358	1.3 (1.1, 1.6)	1.3 (1.1, 1.6)	1.3 (1.1, 1.6)
	4+	9,921	59,519	164	1.8 (1.5, 2.2)	1.5 (1.2, 1.8)	1.5 (1.2, 1.9)
Heart failure	0	36,728	220,480	20	1	1	1
	1	24,229	146,190	26	1.8 (0.97, 3.1)	1.6 (0.88, 3.0)	1.7 (0.89¸ 3.2)
	2	78,961	477,689	56	0.98 (0.58, 1.6)	1.2 (0.67, 2.0)	1.2 (0.68, 2.1)
	3	30,787	186,642	30	1.2 (0.70, 2.2)	1.3 (0.72, 2.4)	1.3 (0.68, 2.4)
	4+	9,921	60,193	12	1.5 (0.70, 3.0)	1.1 (0.52, 2.5)	1.1 (0.48, 2.5)
Pulmonary heart disease and diseases of pulmonary circulation	0	36,728	220,419	54	1	1	1
	1	24,229	146,194	40	1.0 (0.69, 1.6)	1.0 (0.68, 1.6)	0.96 (0.63, 1.5)
	2	78,961	477,543	121	0.86 (0.62, 1.2)	0.88 (0.63, 1.2)	0.81 (0.58, 1.1)
	3	30,787	186,616	49	0.85 (0.58, 1.3)	0.86 (0.58, 1.3)	0.79 (0.52, 1.2)
	4+	9,921	60,154	27	1.4 (0.88, 2.2)	1.2 (0.73, 1.9)	1.2 (0.71, 1.9)

Diseases of arteries, arterioles and capillaries	0	36,728	220,227	75	1	1	1
	1	24,229	146,120	37	0.71 (0.48, 1.1)	0.69 (0.46, 1.0)	0.67 (0.44, 1.0)
	2	78,961	477,447	98	0.53 (0.39, 0.72)	0.55 (0.40, 0.76)	0.57 (0.41, 0.79)
	3	30,787	186,546	42	0.56 (0.38, 0.82)	0.56 (0.37, 0.83)	0.56 (0.37, 0.84)
	4+	9,921	60,159	20	0.81 (0.49, 1.3)	0.80 (0.48, 1.4)	0.78 (0.46, 1.3)

CI: confidence interval; CVD: cardiovascular disease; HR: hazard ratio; IHD: ischemic heart disease.

Model 1 adjusted for age (n= 180,626).
Model 2 adjusted for age, ethnicity, qualifications, income, Townsend deprivation index and family history of cardiovascular disease, smoking, frequency of alcohol intake, frequency of physical activity, body-mass index, diabetes, diastolic blood pressure and systolic blood pressure (n= 176,431).
Model 3 adjusted for all factors in model 2 in addition to age at menarche, menopause, hormone replacement therapy and oral contraceptive use (n=170,418).

eTable 6 The Association Between Parity and Cardiovascular Disease Among Women

Cardiovascular disease outcome	Parity	N	Person years	n events	Model 1 HR (95% CI)	Model 2 HR (95% CI)
CVD	0	36,550	215,128	1,051	1	1
	1	23,828	140,287	870	1.2 (1.1, 1.3)	1.2 (1.1, 1.3)
	2	77,744	458,630	2,779	1.1 (1.0, 1.2)	1.1 (1.0, 1.2)
	3	31,397	185,153	1,251	1.2 (1.1, 1.3)	1.2 (1.1, 1.3)
	4+	11,107	65,188	520	1.4 (1.2, 1.5)	1.2 (1.1, 1.3)
IHD	0	36,550	219,045	112	1	1
	1	23,828	143,398	120	1.5 (1.2, 1.9)	1.4 (1.1, 1.8)
	2	77,744	469,193	327	1.1 (0.88, 1.4)	1.1 (0.89, 1.4)
	3	31,397	189,824	153	1.2 (0.94, 1.5)	1.1 (0.86, 1.4)
	4+	11,107	67,031	86	1.8 (1.4, 2.4)	1.4 (1.0, 1.9)
Cerebrovascular disease	0	36,550	219,244	76	1	1
	1	23,828	143,739	45	0.82 (0.57, 1.2)	0.80 (0.55, 1.2)
	2	77,744	469,978	151	0.73 (0.55, 0.97)	0.78 (0.59, 1.1)
	3	31,397	190,196	81	0.91 (0.66, 1.2)	0.91 (0.66, 1.3)
	4+	11,107	67,226	36	1.1 (0.73, 1.6)	0.99 (0.65, 1.5)
Hypertensive disorders	0	36,550	218,393	249	1	1
	1	23,828	142,711	264	1.5 (1.3, 1.8)	1.4 (1.2, 1.7)
	2	77,744	467,457	707	1.1 (0.94, 1.3)	1.1 (0.96, 1.3)
	3	31,397	188,878	371	1.4 (1.1, 1.6)	1.3 (1.1, 1.6)
	4+	11,107	66,594	179	1.8 (1.5, 2.2)	1.5 (1.2, 1.8)
Heart failure	0	36,550	219,420	19	1	1
	1	23,828	143,781	26	1.9 (1.0, 3.4)	1.8 (0.94, 3.3)
	2	77,744	470,309	55	1.0 (0.61, 1.7)	1.2 (0.70, 2.1)
	3	31,397	190,370	27	1.1 (0.63, 2.1)	1.3 (0.69, 2.4)
	4+	11,107	67,315	17	1.9 (0.99, 3.7)	1.5 (0.74, 3.1)
Pulmonary heart disease and diseases of pulmonary circulation	0	36,550	219,354	54	1	1
	1	23,828	143,797	37	0.97 (0.64, 1.5)	0.97 (0.63, 1.5)
	2	77,744	470,158	120	0.86 (0.62, 1.2)	0.89 (0.64, 1.2)
	3	31,397	190,336	50	0.85 (0.57, 1.3)	0.84 (0.57, 1.3)
	4+	11,107	67,282	30	1.4 (0.88, 2.2)	1.2 (0.74, 1.9)

Diseases of arteries, arterioles and capillaries	0	36,550	219,166	74	1	1
	1	23,828	143,715	36	0.71 (0.47, 1.1)	0.69 (0.46, 1.0)
	2	77,744	470,069	97	0.54 (0.40, 0.73)	0.57 (0.41, 0.78)
	3	31,397	190,262	44	0.58 (0.40, 0.85)	0.59 (0.40, 0.88)
	4+	11,107	67,286	21	0.77 (0.47, 1.3)	0.73 (0.43, 1.2)

CI: confidence interval; CVD: cardiovascular disease; HR: hazard ratio; IHD: ischemic heart disease.

Model 1 adjusted for age (n= 180,626).
Model 2 adjusted for age, ethnicity, qualifications, income, Townsend deprivation index and family history of cardiovascular disease, smoking, frequency of alcohol intake, frequency of physical activity, body-mass index, diabetes, diastolic blood pressure and systolic blood pressure (n=176,431).

eTable 7 The Association Between the Number of Live Births with Overall Cardiovascular Disease Among Women Stratified by Country

Model	Country	Number of live births				
		0<br>HR (95% CI)	1<br>HR (95% CI)	2<br>HR (95% CI)	3<br>HR (95% CI)	4 or more<br>HR (95% CI)
Model 1	England (n=160,241)	1	1.2 (1.1, 1.3)	1.1 (1.0, 1.2)	1.2 (1.1, 1.3)	1.3 (1.2, 1.5)
	Wales (n=7,220)	1	1.2 (0.90, 1.5)	1.0 (0.83, 1.3)	1.1 (0.86, 1.4)	1.3 (0.94, 1.4)
	Scotland (n=13,165)	1	1.3 (1.0, 1.7)	1.0 (0.85, 1.2)	0.99 (0.79, 1.2)	1.6 (1.2, 2.1)
Model 2	England (n=156,468)	1	1.2 (1.1, 1.3)	1.1 (1.0, 1.2)	1.2 (1.1, 1.3)	1.2 (1.0, 1.3)
	Wales (n=7,069)	1	1.1 (0.83, 1.4)	1.0 (0.84, 1.3)	1.0 (0.82, 1.3)	1.1 (0.83, 1.6)
	Scotland (n=12,894)	1	1.3 (1.0, 1.6)	1.1 (0.88, 1.3)	1.0 (0.79, 1.3)	1.4 (1.1, 1.9)

CI: confidence interval; HR: hazard ratio.

Model 1: Adjusted for age.

Model 2: Adjusted for age, ethnicity, qualifications, income, Townsend deprivation index and family history of cardiovascular disease, in addition to smoking, frequency of alcohol intake, frequency of physical activity, body-mass index, diabetes, diastolic blood pressure and systolic blood pressure.

The p-value for likelihood ratio test comparing the model with and without the interaction term was 0.570. The interaction was tested in the fully adjusted model.

eTable 8 The Association Between the Number of Children Fathered with Overall Cardiovascular Disease Among Men Stratified by Country

Model	Country	Number of children fathered					
		0	1<br><br>HR (95% CI)	2<br><br>HR (95% CI)	3<br><br>HR (95% CI)	4 or more<br><br>HR (95% CI)	Unknown/Prefer not to answer<br>HR (95% CI)
Model 1	England (n=118,453)	1	1.1 (1.0, 1.2)	0.96 (0.88, 1.0)	1.0 (0.95, 1.1)	1.2 (1.0, 1.3)	1.4 (1.1, 1.9)
	Wales (n=5,477)	1	0.92 (0.70, 1.2)	1.0 (0.83, 1.2)	1.1 (0.85, 1.4)	1.3 (0.98, 1.8)	1.1 (0.49, 2.5)
	Scotland (n=9,329)	1	1.2 (0.97, 1.5)	0.94 (0.78, 1.1)	1.0 (0.84, 1.3)	0.95 (0.71, 1.3)	1.4 (0.74, 2.8)
Model 2	England (n= 115,078)	1	1.1 (1.0, 1.2)	1.0 (0.94, 1.1)	1.1 (1.0, 1.2)	1.1 (0.99, 1.3)	1.1 (0.83, 1.6)
	Wales (n= 5,356)	1	0.91 (0.69, 1.2)	1.0 (0.84, 1.3)	1.1 (0.86, 1.4)	1.3 (0.93, 1.7)	0.85 (0.35, 2.1)
	Scotland (n= 9,116)	1	1.2 (0.98, 1.6)	1.0 (0.84, 1.2)	1.2 (0.93, 1.4)	0.93 (0.69, 1.3)	1.5 (0.74, 2.8)

CI: confidence interval; HR: hazard ratio.

Model 1: Adjusted for age.

Model 2: Adjusted for age, ethnicity, qualifications, income, Townsend deprivation index and family history of cardiovascular disease, in addition to smoking, frequency of alcohol intake, frequency of physical activity, body-mass index, diabetes, diastolic blood pressure and systolic blood pressure.

The p-value for likelihood ratio test comparing the model with and without the interaction term was 0.530. The interaction was tested in the fully adjusted model.

eTable 9 The Association Between the Number of Live Births with Overall Cardiovascular Disease Among Women Stratified by Age at Recruitment

Model	Age	Number of live births				
		0	1<br><br>HR (95% CI)	2<br><br>HR (95% CI)	3<br><br>HR (95% CI)	4 or more<br><br>HR (95% CI)
Unadjusted	<=48 (n=46,146)	1	1.2 (0.97,1.4)	1.1 (0.93, 1.3)	1.3 (1.0, 1.5)	1.6 (1.2, 2.2)
	49-55 (n=44,368)	1	1.1 (0.92, 1.4)	1.2 (1.0, 1.4)	1.2 (0.99, 1.4)	1.6 (1.3, 2.0)
	56-62 (n=52,008)	1	1.3 (1.1, 1.5)	1.1 (0.92, 1.2)	1.1 (0.97, 1.3)	1.3 (1.0, 1.5)
	63 and over (n=38,104)	1	1.2 (1.0, 1.4)	1.1 (0.93, 1.2)	1.2 (0.99, 1.4)	1.3 (1.1, 1.6)
Adjusted	<=48 (n= 45,176)	1	1.2 (0.94, 1.4)	1.2 (0.99, 1.4)	1.3 (1.0, 1.6)	1.4 (1.0, 1.9)
	49-55 (n= 43,449)	1	1.1 (0.86, 1.3)	1.2 (1.0, 1.4)	1.2 (0.97, 1.4)	1.3 (1.0, 1.7)
	56-62 (n= 50,847)	1	1.3 (1.1, 1.5)	1.0 (0.91, 1.2)	1.1 (0.91, 1.3)	1.1 (0.88, 1.4)
	63 and over (n=36,959)	1	1.2 (0.97, 1.4)	1.1 (0.93, 1.2)	1.2 (0.98, 1.3)	1.2 (0.95, 1.4)

CI: confidence interval; HR: hazard ratio.

Adjusted for age, ethnicity, qualifications, income, Townsend deprivation index and family history of cardiovascular disease, in addition to smoking, frequency of alcohol intake, frequency of physical activity, body-mass index, diabetes, diastolic blood pressure and systolic blood pressure.

The p-value for likelihood ratio test comparing the model with and without the interaction term was 0.323. The interaction was tested in the fully adjusted model.

eTable 10 The Association Between the Number of Children Fathered With Overall Cardiovascular Disease Among Men Stratified by Age at Recruitment

Model	Age	Number of children fathered					
		0	1<br><br>HR (95% CI)	2<br><br>HR (95% CI)	3<br><br>HR (95% CI)	4 or more<br><br>HR (95% CI)	Unknown/Prefer not to answer<br>HR (95% CI)
Unadjusted	<=48 (n= 36,683)	1	1.1 (0.93, 1.4)	1.0 (0.85, 1.2)	1.2 (0.95, 1.4)	1.4 (1.1, 1.8)	0.75 (0.34, 1.7)
	49-55 (n= 30,624)	1	1.0 (0.85, 1.2)	0.83 (0.72, 0.96)	0.98 (0.82, 1.2)	1.0 (0.80, 1.3)	1.3 (0.75, 2.2)
	56-62 (n= 36,469)	1	1.2 (1.0, 1.4)	1.1 (0.94, 1.2)	1.2 (1.0, 1.4)	1.3 (1.1, 1.5)	1.7 (1.1, 2.5)
	63 and over (n=29,483)	1	1.1 (0.93, 1.3)	0.99 (0.88, 1.1)	1.0 (0.90, 1.2)	1.1 (0.95, 1.3)	1.5 (0.98, 2.3)
Adjusted	<=48 (n= 35,625)	1	1.2 (0.94, 1.4)	1.1 (0.89, 1.3)	1.2 (0.94, 1.5)	1.3 (0.97, 1.7)	0.52 (0.19, 1.4)
	49-55 (n= 29,833)	1	1.0 (0.86, 1.3)	0.89 (0.77, 1.0)	1.0 (0.84, 1.2)	1.0 (0.79, 1.3)	0.94 (0.48, 1.8)
	56-62 (n= 35,588)	1	1.1 (0.97, 1.3)	1.1 (0.97, 1.2)	1.2 (1.1, 1.4)	1.3 (1.0, 1.5)	1.4 (0.87, 2.1)
	63 and over (n=28,504)	1	1.1 (0.95, 1.3)	1.1 (0.93, 1.2)	1.1 (0.96, 1.3)	1.1 (0.90, 1.3)	1.4 (0.91, 2.3)

CI: confidence interval; HR: hazard ratio.

Adjusted for age, ethnicity, qualifications, income, Townsend deprivation index and family history of cardiovascular disease, in addition to smoking, frequency of alcohol intake, frequency of physical activity, body-mass index, diabetes, diastolic blood pressure and systolic blood pressure.

The p-value for likelihood ratio test comparing the model with and without the interaction term was 0.591. The interaction was tested in the fully adjusted model.

eTable 11 The Association Between the Number of Live Births with Overall Cardiovascular Disease Among Women Stratified by Year of Birth

Model	Year of birth	Number of live births				
		0	1<br><br>HR (95% CI)	2<br><br>HR (95% CI)	3<br><br>HR (95% CI)	4 or more<br><br>HR (95% CI)
Model 1	<1947 (n=44,998)	1	1.3 (1.1, 1.5)	1.1 (0.95, 1.2)	1.1 (0.99, 1.3)	1.3 (1.1, 1.6)
	1947 -1952 (n=44,780)	1	1.2 (1.0, 1.5)	1.1 (0.92, 1.2)	1.1 (0.95, 1.3)	1.2 (0.91, 1.5)
	1953-1959 (n= 44,244)	1	1.1 (0.91, 1.3)	1.1 (0.93, 1.3)	1.2 (0.96, 1.4)	1.4 (1.1, 1.9)
	1960 and later (n=46,604)	1	1.2 (0.99, 1.5)	1.2 (0.97, 1.4)	1.3 (1.0, 1.5)	1.7 (1.3, 2.3)
Model 2	<1947 (n= 43,682)	1	1.2 (1.0, 1.4)	1.1 (0.94, 1.2)	1.1 (0.98, 1.3)	1.2 (1.0, 1.4)
	1947 -1952 (n= 43,791)	1	1.2 (1.0, 1.4)	1.1 (0.92, 1.2)	1.1 (0.91, 1.3)	1.0 (0.81, 1.3)
	1953-1959 (n= 43,353)	1	1.1 (0.86, 1.3)	1.1 (0.96, 1.3)	1.2 (0.96, 1.4)	1.2 (0.94, 1.6)
	1960 and later (n= 45,605)	1	1.2 (0.96, 1.5)	1.2 (1.0, 1.5)	1.3 (1.0, 1.6)	1.5 (1.1, 2.0)

CI: confidence interval; HR: hazard ratio.

Model 1: Adjusted for age
Model 2: Adjusted for age, ethnicity, qualifications, income, Townsend deprivation index and family history of cardiovascular disease, in addition to smoking, frequency of alcohol intake, frequency of physical activity, body-mass index, diabetes, diastolic blood pressure and systolic blood pressure.

The p-value for likelihood ratio test comparing the model with and without the interaction term was 0.642. The interaction was tested in the fully adjusted model.

eTable 12 The Association Between the Number of Children Fathered With Overall Cardiovascular Disease Among Men Stratified by Year of Birth

Model	Year of birth	Number of children fathered					
		0	1<br><br>HR (95% CI)	2<br><br>HR (95% CI)	3<br><br>HR (95% CI)	4 or more<br><br>HR (95% CI)	Unknown/Prefer not to answer<br>HR (95% CI)
Model 1	<1947 (n=34,282)	1	1.1 (0.94, 1.3)	1.0 (0.89, 1.1)	1.1 (0.93, 1.2)	1.2 (0.99, 1.4)	1.5 (1.0, 2.1)
	1947 -1952 (n=31,361)	1	1.1 (0.96, 1.4)	1.0 (0.88, 1.2)	1.2 (1.0, 1.4)	1.2 (0.98, 1.5)	1.5 (0.95, 2.4)
	1953-1959 (n=30,598)	1	1.0 (0.87, 1.3)	0.83 (0.71, 0.96)	0.91 (0.76, 1.1)	0.95 (0.74, 1.2)	1.5 (0.94, 2.5)
	1960 and later (n=37,018)	1	1.2 (0.94, 1.4)	0.99 (0.83, 1.2)	1.2 (0.94, 1.4)	1.4 (1.1, 1.9)	0.65 (0.27, 1.6)
Model 2	<1947 (n= 33,190)	1	1.1 (0.96, 1.3)	1.1 (0.94, 1.2)	1.1 (0.98, 1.3)	1.1 (0.95, 1.3)	1.4 (0.92, 2.1)
	1947 -1952 (n= 30,608)	1	1.1 (0.94, 1.4)	1.1 (0.95, 1.3)	1.3 (1.1, 1.5)	1.2 (0.99, 1.5)	1.2 (0.71, 2.1)
	1953-1959 (n= 29,826)	1	1.1 (0.87, 1.3)	0.89 (0.76, 1.0)	0.95 (0.79, 1.2)	0.93 (0.72, 1.2)	1.1 (0.62, 2.1)
	1960 and later (n= 35,926)	1	1.2 (0.95, 1.5)	1.1 (0.90, 1.3)	1.2 (0.95, 1.5)	1.3 (1.0, 1.8)	0.42 (0.13, 1.3)

CI: confidence interval; HR: hazard ratio.

Model 1: Adjusted for age.
Model 2: Adjusted for age, ethnicity, qualifications, income, Townsend deprivation index and family history of cardiovascular disease, in addition to smoking, frequency of alcohol intake, frequency of physical activity, body-mass index, diabetes, diastolic blood pressure and systolic blood pressure.

The p-value for likelihood ratio test comparing the model with and without the interaction term was 0.361. The interaction was tested in the fully adjusted model.

eTable 13 The Association Between Number of Offspring and Overall Cardiovascular Disease Comparing the Use of Calendar Time and Age as Axis for the Cox Model

Gender	Number of offspring	Calendar time as axis Adjusted HR (95% CI)[a]	Age as time axis Adjusted HR (95% CI)[b]
Women	0	1	1
	1	1.2 (1.1, 1.3)	1.2 (1.1, 1.3)
	2	1.1 (1.0, 1.2)	1.2 (1.1, 1.3)
	3	1.2 (1.1, 1.3)	1.2 (1.1, 1.4)
	4+	1.2 (1.1, 1.4)	1.3 (1.2, 1.5)
Men	0	1	1
	1	1.1 (1.0, 1.2)	1.2 (1.1, 1.3)
	2	1.0 (0.96, 1.1)	1.1 (1.0, 1.2)
	3	1.1 (1.0, 1.2)	1.2 (1.1, 1.3)
	4+	1.1 (1.0, 1.3)	1.2 (1.1, 1.4)
	Unknown/ prefer not to answer	1.2 (0.88, 1.5)	1.2 (0.90, 1.6)

CI: confidence interval; HR: hazard ratio.

[a] Adjusted for age, ethnicity, qualifications, income, Townsend deprivation index and family history of cardiovascular disease, in addition to smoking, frequency of alcohol intake, frequency of physical activity, body-mass index, diabetes, diastolic blood pressure and systolic blood pressure.

[b] Adjusted for ethnicity, qualifications, income, Townsend deprivation index and family history of cardiovascular disease, in addition to smoking, frequency of alcohol intake, frequency of physical activity, body-mass index, diabetes, diastolic blood pressure and systolic blood pressure.

eTable 14 Association between Known Risk Factors and Overall Cardiovascular Disease among Women and Men

	Women (n=180,626)	Men (n=133,259)
	HR (95% CI)	HR (95% CI)
Age	1.1 (1.1, 1.1)	1.1 (1.1, 1.1)
Smoking		
No	1	1
Previous	1.2 (1.1, 1.3)	1.2 (1.1, 1.2)
Current	1.6 (1.4, 1.7)	1.6 (1.5, 1.7)
Alcohol intake frequency		
Daily or almost daily	1	1
Three or four times a week	0.93 (0.86, 1.0)	0.90 (0.78, 1.0)
Once or twice a week	1.1 (0.99, 1.2)	1.0 (0.87, 1.1)
One to three times a month	1.2 (1.1, 1.3)	0.97 (0.81, 1.2)
Special occasions only	1.3 (1.2, 1.4)	1.4 (1.2, 1.7)
Never	1.5 (1.4, 1.7)	1.3 (1.1, 1.6)
Body-mass index (kg/m^2)		
Continuous	1.1 (1.1, 1.1)	1.0 (1.0, 1.1)
Underweight	1.5 (1.2, 2.0)	1.5 (1.0, 2.2)
Normal Weight	1	1
Overweight	1.4 (1.3, 1.5)	1.2 (1.1, 1.2)
Obese	1.9 (1.8, 2.0)	1.5 (1.4, 1.6)
Diastolic blood pressure (mmHG)	1.0 (1.0, 1.0)	1.0 (1.0, 1.0)
Systolic blood pressure (mmHG)	1.0 (1.0, 1.0)	1.0 (1.0, 1.0)
Diabetes diagnosed by a doctor		
No	1	1
Yes	2.1 (1.8, 2.4)	2.0 (1.8, 2.3)

CI: confidence interval; HR: hazard ratio.

eTable 15 The Association Between Number of Offspring and All-Cause Mortality Among Women and Men

Outcome	Number of offspring	Model 1 HR (95% CI)	Model 2 HR (95% CI)
Women	0	1	1
	1	0.88 (0.80, 0.97)	0.84 (0.75, 0.93)
	2	0.71 (0.65, 0.76)	0.76 (0.70, 0.82)
	3	0.74 (0.67, 0.81)	0.75 (0.68, 0.82)
	4+	0.92 (0.82, 1.0)	0.78 (0.69, 0.88)
	1 or more	1	1
	0	1.3 (1.2, 1.4)	1.3 (1.2, 1.4)
Men	0	1	1
	1	0.87 (0.81, 0.95)	0.94 (0.87, 1.02)
	2	0.66 (0.62, 0.70)	0.81 (0.76, 0.86)
	3	0.70 (0.65, 0.75)	0.81 (0.75, 0.88)
	4+	0.90 (0.82, 0.98)	0.86 (0.79, 0.95)
	Do not know/prefer not to say	1.4 (1.2, 1.7)	1.1 (0.93, 1.4)
	1 or more	1	1
	0	1.4 (1.3, 1.4)	1.2 (1.1, 1.3)

CI: confidence interval; HR: hazard ratio.

Model 1 Adjusted for age.
Model 2 Adjusted for ethnicity, qualifications, income, Townsend deprivation index and family history of cardiovascular disease, in addition to smoking, frequency of alcohol intake, frequency of physical activity, body-mass index, diabetes, diastolic blood pressure and systolic blood pressure.
